# AChE-activity in critically ill patients with suspected septic encephalopathy: a prospective, single-centre study

**DOI:** 10.1186/s12871-020-01204-6

**Published:** 2020-11-17

**Authors:** Benedikt Zujalovic, Benjamin Mayer, Sebastian Hafner, Florian Balling, Eberhard Barth

**Affiliations:** 1 Department of Anesthesiology, Section Interdisciplinary Surgical Intensive Care, University Hospital Ulm, Albert-Einstein-Allee 23, 89081 Ulm, Germany; 2 Ulm University, Institute of Epidemiology and Medical Biometry, Schwabstraße 13, 89075 Ulm, Germany

**Keywords:** Septic associated encephalopathy, Cholinergic dysfunction, Acetylcholinesterase-activity, Delirium, Cognitive dysfunction

## Abstract

**Background:**

Up to 70% of septic patients develop a diffuse brain dysfunction named “septic associated encephalopathy” which is often solely based on clinical impressions. However, the diagnosis of septic associated encephalopathy is outcome-relevant due to an increase in mortality in these patients. Neuroinflammation as well as a disturbance of cholinergic transmission are assumed to be the causes of both delirium and septic associated encephalopathy. An alteration in cholinergic activity can be objectified by measuring the erythrocytic acetylcholinesterase-activity. Single-point measurements of acetylcholinesterase-activity are of limited value because individual and dynamic changes over time have to be anticipated. Therefore, the hypothesis should be tested whether a longitudinal analysis of acetylcholinesterase-activity in critically ill patients can help to diagnose a suspected septic-associated encephalopathy and whether acetylcholinesterase-activity differs in comparison to non-septic patients.

**Methods:**

In this prospective, observational, single-center study, 175 patients (45 with sepsis, 130 without sepsis) were included. All patients were admitted to the surgical Intensive Care Unit of the University hospital Ulm, Germany. Patients were examined daily for the presence of delirium using the CAM-ICU. Daily measurement of the acetylcholinesterase-activity was performed in all patients. The possible time-dependent change in acetylcholinesterase-activity was analyzed with a linear regression model considering repeated measurements. Using a time-adjusted model further factors able to affect AChE-activity were investigated. For nonparametric distributions quantitative data were compared using Wilcoxon matched-pairs test. For analysis of independent samples the Mann-Whitney test was performed.

**Results:**

About 90% of septic patients with suspected septic associated encephalopathy exhibited a statistically significant time-dependent in- or decrease in acetylcholinesterase-activity over a period of at least 5 consecutive days.

**Conclusion:**

Longitudinal measurement of acetylcholinesterase-activity over several consecutive days revealed a change from baseline only in septic patients with suspected septic-associated encephalopathy. Therefore, longitudinal measurement of acetylcholinesterase-activity is able to diagnose septic associated encephalopathy in septic patients with delirious symptoms.

**Trial registration:**

Retrospectively registered at German Clinical Trials Register, registration number DRKS00020542, date of registration: January 27, 2020.

## Background

In accordance to the third international consensus definition for sepsis and shock (Sepsis-3), sepsis is defined as a syndrome of physiological, pathological and biochemical changes due to an infection [1, 2]. As a result of systemic inflammation up to 70% of septic patients develop a diffuse brain dysfunction called “septic associated encephalopathy (SAE)”, which can be classified under the generic term delirium [3, 4]. Septic patients with delirium have increased mortality compared to septic patients without cognitive impairment. Therefore, central nervous system (CNS) involvement in septic patients appears to be an outcome-relevant issue [5–7]. The underlying pathophysiological causes of SAE appear to be different from non-septic delirium and should be considered more closely. The occurrence of delirium symptoms in critically ill patients is multifactorial. In particular, augmented permeability of the blood-brain barrier (BBB) due to systemic inflammation is a major pathophysiological mechanism in both, delirium and SAE [7–10]. Beside the loss of the integrity of the BBB, pro-inflammation leads to microvascular-endothelial dysfunction and excessive activation of inflammatory cells of the CNS. This results in a gradual loss of (cholinergic) neurons of the forebrain [6, 11–13]. The resulting lack of acetylcholine (Ach) leads to attention deficits, cognitive impairment and memory disorders [14]. In addition to cognitive impairment, the disruption of cholinergic activity has direct effects on the vagal-mediated immune response. The interaction between CNS and peripheral immune response, the so-called cholinergic-anti-inflammatory pathway, plays an important role in the control of inflammation. A disorder of the cholinergic-anti-inflammatory pathway is discussed as a pathophysiological cause of SAE [3, 11–13, 15, 16]. The cholinergic-anti-inflammatory pathway can be stimulated by the application of indirect parasympathomimetics, with improved immune response in experimental sepsis models [17–19]. Thus, the central cholinergic metabolism in septic patients plays a much more important role than previously assumed [20–23]. The binding of Ach to CNS-localized nicotinic acetylcholine receptors modulates the neuronal level of excitation as well as learning and memory competence [7]. Disruption of central cholinergic homeostasis by systemic inflammation can manifest clinically as SAE [8, 12]. Since Ach cannot be measured directly due to rapid degradation in the synaptic cleft, a surrogate parameter for central-cholinergic metabolism is required. The erythrocytic acetylcholinesterase-activity (AChE-activity) has proven to be suitable. The AChE-activity is characterized by its high affinity for the transmitter acetylcholine, its inhibition by high Ach-concentrations and its low affinity for non-cholinesters [24]. The AChE-activity in the erythrocytic plasma corresponds to the AChE-activity in cerebrospinal fluid (CSF) and thus seems to be a suitable surrogate parameter of cholinergic transmission in the CNS [25]. Using point-of-care diagnostics, AChE-activity can be measured within minutes and disturbances in central cholinergic transmission can be objectified. If indirect centrally acting parasympathomimetic drugs are considered for the treatment of SAE/delirium, point-of-care diagnostics can be used to monitor this therapy [26, 27]. Due to the increasing knowledge about the role of cholinergic transmission in sepsis, neuroinflammation and concomitant cognitive disorders, this study investigated whether AChE-activity is altered in septic patients with SAE/delirium. Moreover, it was of interest to what extent AChE-activity in septic patients with pronounced “inflammatory load” differs from non-septic, critically-ill patients with and without delirium.

## Methods

The prospective, observational, single-center study was conducted in the observation period from 03/2017 until 03/2018 (positive vote of the local ethics committee, Trial-Code No. 363/16) in the interdisciplinary surgical intensive care unit (ICU), University Hospital of Ulm, Germany. The data evaluation took place in the period from 05/2018 to 06/2019. The study was retrospectively registered at the German Clinical Trials Register (DRKS-ID: DRKS00020542). The study protocol was in accordance to the Declaration of Helsinki ethical guidelines. All patients or their legal designees signed written informed consent to take part in this study.

Inclusion criteria:

- Age ≥ 18 years
- need for intensive care treatment due to an emergency or elective surgery
- expected stay on the ICU for at least 24 h
- ≥ 2 values of AChE-activity
- ability to understand and speak the German language

Exclusion criteria:

- Age < 18 years
- < 2 consecutive measurement values of the AChE-activity
- missing written informed consent

The following patient-related data were collected during the stay on the ICU:

- Age at enrollment
- gender
- ICU length of stay
- disease severity scores (SAPS II, TISS-28)
- Richmond Agitation Sedation Scale (RASS)
- Confusion assessment method for the intensive care unit (CAM-ICU)
- primary reason for ICU admission
- several laboratory parameters (subsumed under the SOFA-Score, TISS-28, SAPS II)
- vital signs (heart rate, blood pressure, respiratory rate)

For characterization of the heterogenous study population the relevant baseline data (demographic data, primary reason for ICU admission) were collected. The severity of illness was quantified using the Simplified Acute Physiology Score (SAPS II), as well as the Therapeutic Intervention Scoring System (TISS-28). The TISS-28 records the daily condition of the patient by recording the therapeutic, diagnostic and nursing measures [28]. Both scoring systems display the severity of illness in critically ill patients and thus allow comparison within the framework of studies.

### Definition of sepsis

Sepsis and septic shock respectively were diagnosed according to the third international consensus definitions for sepsis and septic shock (Sepsis-3) in 2016 [2]. Patients were classified as septic if they met the criteria of the Sepsis-3 definition at admission or within 24 h after admission to the intensive care unit [1, 2]. Beside the sequential organ failure assessment-Score (SOFA-Score), inflammatory parameters (C-reactive Protein (CRP), Procalcitonin (PCT), white blood cells) were collected.

### Definition of delirium, cognitive dysfunction and septic associated encephalopathy

#### Definition of delirium

Delirium is a common syndrome on ICU and will be divided into the hypoactive-, the hyperactive- and the mixed- type [29]. In the present study, the delirium was diagnosed by using the CAM-ICU, German Version. It was performed by trained personal (nurses and physicians) at least once every 8 hours. Delirium was primarily diagnosed in all patients with a positive CAM-ICU test result, regardless of the presence of sepsis.

#### Definition of cognitive dysfunction

Critically ill patients with a lower vigilance level RASS < − 3 cannot be examined with current delirium screening instruments such as the CAM-ICU. Those patients cannot comply simple prompts such as show one’s teeth and tongue, squeezing one’s hand e.g. These patients may also show up by an uncoordinated adaption to the respirator, agitation and the inability to reach a sufficient level of contact. The limitations mentioned for performing the CAM-ICU are often observed in critically ill patients with intracranial bleeding or neurocognitive disorders. For these patients the somewhat controversial term “cognitive dysfunction” has been chosen, which should be interpreted in a purely descriptive manner [27].

#### Definition of septic associated encephalopathy

In septic patients with delirious symptoms (CAM-ICU positive) septic-associated encephalopathy must be considered for differential diagnosis. However, before SAE can be diagnosed, structural changes of the brain due to craniocerebral trauma or ischemia as well as adverse drug reactions must be excluded [30, 31]. Validated delirium screening tools like the CAM-ICU have proven to be suitable for diagnosing SAE [28]. Aware that the CAM-ICU can support the suspected diagnosis of SAE, but cannot prove it, the following consideration should be taken into account: Septic patients in whom the CAM-ICU cannot be reliably determined should not be assigned to the category “SAE”. These patients are referred to as septic patients with cognitive dysfunction. Patients in the present study were suspected to have SAE under the following criteria: Diagnosed sepsis with concomitant delirious symptoms and positive CAM-ICU test result.

Cognitive dysfunction and septic-associated encephalopathy are often summarized under the generic term delirium. However, they are different from each other in significant aspects.

### AChE-activity measurements

Since acetylcholine cannot be measured directly due to its rapid enzymatic degradation by acetylcholinesterase, an appropriate surrogate parameter for the (central) cholinergic acetylcholine metabolism is necessary. The erythrocytic acetylcholinesterase activity has proven to be a suitable surrogate parameter in numerous studies [26, 27, 32]. One ethylenediamine tetraacetic acid (EDTA)-Blood sample (1 ml) was collected once daily over a period of maximum 6 days at 5:00 a.m. The first blood sample was taken in the morning after admission on the ICU, labeled as “day 1”. Between 7:00 and 12:00 a.m. the erythrocyte AChE-activity was determined by using LISA-ChE® (Dr. F. Koehler Chemie GmbH, Germany), a point-of-care testing device. The measurement of the AChE-activity is based on the modified Ellman method, a colorimetric method, improved by Worek et al. [26]. The reference values of AChE-activity ranges from 26.7 U/gHb until 50.9 U/gHb [26, 27]. Studies on the re-evaluation of reference values of AChE-activity in intensive care patients are still missing up to now. Moreover, the inter- and intraindividual variability and the time-dependent changes in AChE-activity in critically ill patients have not yet been adequately investigated. The primary endpoint of this study was to investigate whether AChE-activity is altered in septic patients with suspected SAE compared to non-septic patients with and without delirium. The secondary endpoint was to investigate whether AChE-activity is capable of differentiating between SAE and other causes of delirium in critically ill patients.

### Sample size calculation and power analysis

Previously published data on AChE-activity in delirious intensive care patients have shown that even in small case numbers a statistically significant change in AChE-activity can be detected [27, 32]. Own data were used as a pilot data set for the sample size planning of this study. In addition, the following considerations were taken into account: the average number of intensive care patients in the interdisciplinary surgical intensive care unit, University hospital Ulm, is about 550 patients per year. The prevalence of sepsis in German intensive care units was about 12.4% (sepsis) and 11.0% (severe sepsis and septic shock) in the observation period. A total number of 200 patients was calculated for the prospective observational study (GPower 3.1). After completion of the patient recruitment, which was limited for a maximum period of 12 months in the study plan, the patients were divided into a septic and a non-septic group. Subsequently, a post-hoc power calculation was performed. In detail, a simulation-based approach has been used in order to assess the power associated to a longitudinal AChE-activity regression model including the time point of measurement, group status of the patient (septic vs. non-septic) as well as the corresponding interaction term. This analysis was conducted by means of the SIMR package in R (version 3.6.1). Based on the sample size of about 40 patients in the smaller (septic) subgroup, the simulation shows an empirical significance of about 60%. Considering the statistically significant results, the empirical power is calculated in a range between 60 and 80% despite the large difference.

### Statistical analysis

Data were collected in Microsoft Excel 2010 (Microsoft Corp., Redmond, WA) and analyzed by using GraphPad PRISM, Version 5 for Windows and SAS Version 9.4.

AChE-activity was analyzed over the course of time by using a linear regression model accounting for repeated measures. The AChE-activity was defined as the dependent variable and the duration of measurement (a maximum of six consecutive days) was defined as the continuous independent predictor of primary interest. Using a time adjusted model, the effect of further possible predictors of AChE-activity was analyzed.

Quantitative data were expressed as median, minimum and maximum and, for nonparametric distributions, were compared using the Wilcoxon matched-pairs test. For the analysis of the independent samples, we used the Mann-Whitney test. All results reported have to be interpreted in an exploratory manner, since we did not adjust the *p*-values for multiple testing.

## Results

During the observation period, 241 potentially eligible patients were admitted to the surgical intensive care unit at the University Hospital of Ulm, Germany. Of these, 66 patients had to be excluded for the following reasons: In 38 patients the number of AChE-activities measured was less than 2 readings (length of stay in intensive care unit < 24 h - 10 patients died, 28 patients were transferred to IMC). In 10 patients the data set was incomplete. In 18 patients, written consent was not available at the time of data analysis. Finally, a total of 175 patients were included in the further analysis. An imbalance in gender distribution favoring the male sex occurred in both groups (Table 1). Patients with sepsis had a longer stay in the intensive care unit and higher disease severity scores compared to non-septic patients. The hypoactive course of delirium was more common in septic patients with suspected SAE than in non-septic delirious patients (Table 1).

**Table 1:** Characteristics of patient population

Variable	Patients *n* = 175	
	non-septic patients *n* = 130	septic patients *n* = 45
**Age at enrollment**		
Median (min., max.)	64.0 (20.0–95.0)	61.0 (30.0–91.0)
**Gender, n (%)**		
Male	89.0 (68.5)	33 (73.3)
Female	41.0 (31.5)	12 (26.7)
**ICU – LOS - days**		
Median (min., max.)	8 (1.0–86.0)	14.0 (1.0–87.0)
**Overall mortality, n (%)**	10 (7.7)	22 (48.9)
**Disease severity scoring**		
SAPS II		
Median (min., max.,)	26.8 (9.8–54.5)	35.4 (7.0–58.1)
TISS-28		
Median (min., max.,)	10.3 (4.0–30.2)	18.6 (5.0–29.9)
**Delirium n (%)**	36 (27.7)	40 (88.9) DD SAE
• Hypoactive	• 12 (33.3)	• 35 (87.5)
• Hyperactive	• 5 (13.9)	• 1 (2.5)
• Mixed-form	• 19 (52.8)	• 4 (10.0)
**Cognitive dysfunction n (%)**	24 (18.5)	1 (2.2)
**Sedation until decease n (%)**	–	3 (6.7)
**Permanently CAM-ICU negative n (%)**	–	1 (2.2)
**Primary reason for ICU admission, n (%)**		
Neurosurgery & brain haemorrhage	40 (30.8)	4 (8.9)
Abdominal surgery	28 (21.5)	20 (44.4)
Trauma surgery	20 (15.4)	3 (6.7)
Cardiac surgery	15 (11.5)	1 (2.2)
Vascular surgery	12 (9.2)	5 (11.1)
Thoracic surgery	11 (8.5)	4 (8.9)
Respiratory failure	4 (3.1)	5 (11.1)
Haemato-oncology	–	2 (4.4)
Urinary system	–	1. (2.2)

*ICU* Intensive Care Unit, *LOS* Length of stay, *SAPS II* Simplified Acute Physiology Score II, *TISS-28* Therapeutic Intervention Scoring System 28, *CAM-ICU* Confusion Assessment Method for the Intensive Care Unit, *SAE* Septic Associated Encephalopathy,

### Results - non-septic patient group

One hundred thirty patients were classified as non-septic. Of these, 89 were male, 41 female. The mean age of the patients was 64 years. The median length of stay on the ICU was 8 days. Ten of the one hundred thirty patients died during the observation period. Thirty-six patients had a delirium (CAM-ICU positive). Related to the delirium subtypes, 12 patients had a hypoactive, 5 a hyperactive delirium. Nineteen patients showed the appearance of a mixed form with hypo- and hyperactive parts. In 24 of the non-septic patients, the CAM-ICU could not be performed. These patients were given the term “cognitive dysfunction”.

**Fig. 1:**
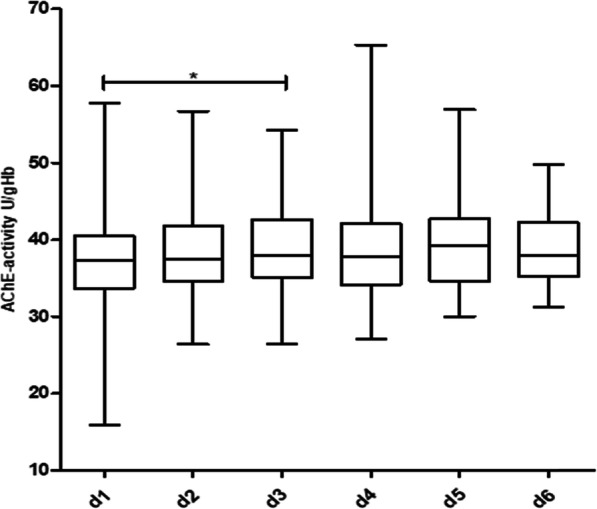
Course of AChE-activity in non-septic patients over a period of 6 days. Statistically significant difference between day 1 and day 3 (*p* = 0.03), calculated with Wilcoxon matched-pairs test. Number of patients per day: d 1: *n* = 130, d 2: n = 130, d 3: *n* = 99, d 4: *n* = 74, d 5: *n* = 69, d 6: *n* = 56

**Fig. 2:**
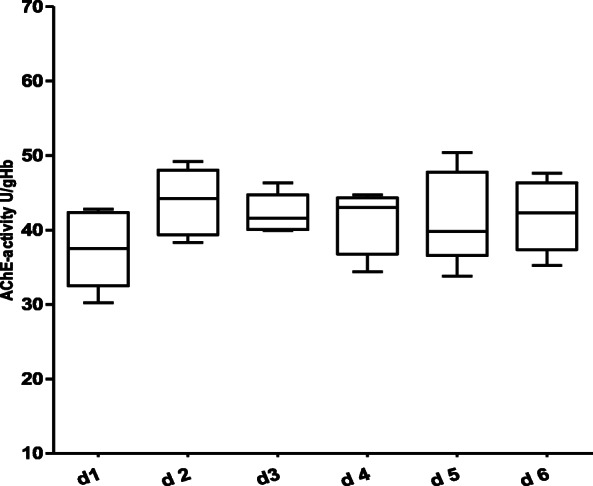
Course of AChE-activity in non-septic, CAM-ICU positive patients over a 6-day observation period with a trend towards an increase of the AChE-activity (no statistically significance difference over the course of time between day 1 until day 6 was observed – Wilcoxon matched-pairs test). Number of patients per day: d 1: *n* = 5, d 2: *n* = 5, d 3: *n* = 5, d 4: *n* = 5, d 5: *n* = 5, d 6: *n* = 5

**Fig. 3:**
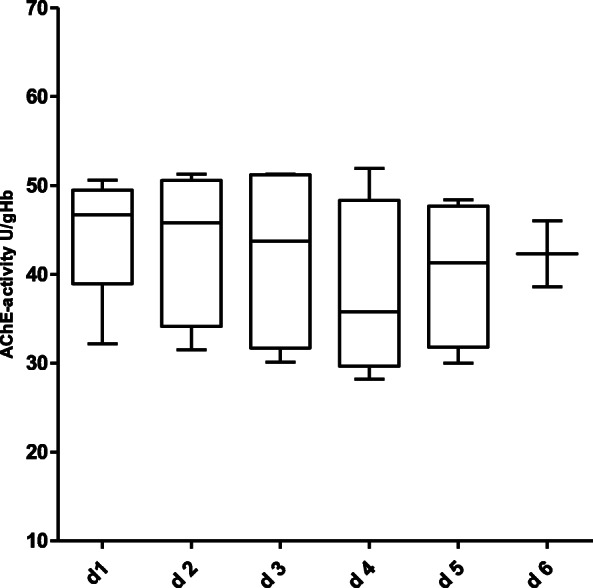
Course of AChE-activity in non-septic, CAM-ICU positive patients over a 6-day observation period with a trend towards a decrease of the AChE-activity (no statistically significance difference over the course of time between day 1 until day 6 was observed – Wilcoxon matched-pairs test). Number of patients per day: d 1: *n* = 5, d 2: *n* = 5, d 3: *n* = 4, d 4: *n* = 4, d 5: *n* = 4, d 6: *n* = 2

Figure 1 shows the course of AChE-activity in the 130 non-septic patients over a period of 6 days. With the exception of a statistically significant increase between day 1 and day 3 (Wilcoxon matched pairs test, *p* = 0.03), no relevant change in AChE-activity over the further observation period was detected. Figures 2 and 3 show the course of AChE-activity in 10 delirious non-septic patients. Five of these patients showed a non-significant increase (Fig. 2) and another five patients a non-significant decrease (Fig. 3) in AChE-activity of at least 10% compared to the baseline at admission to the ICU. The remaining 26 non-septic patients with positive CAM-ICU displayed no change at all in the AChE activity. Therefore, the corresponding box plots were not shown separately.

### Results - septic patient group

Forty-five of one hundred seventy-five patients were septic. Thirty-three patients were male, 12 female. The mean age of the septic patients was 61 years. The median length of stay in the ICU was 14 days. Twenty-two of the septic patients deceased during the observation period. Forty of the septic-patients suffered from delirium. Consequently, these patients were suspected to have been diagnosed with “SAE”. In a septic patient, the CAM-ICU was not feasible due to a cerebral hemorrhage. This patient was designated as a septic patient with cognitive dysfunction. Three patients were under permanent sedation until death and could not be examined for the presence of delirium. One septic patient never had a delirium during the intensive care.

All septic patients showed a change in AChE-activity, corresponding to a 10% increase or decrease from baseline.

**Fig. 4:**
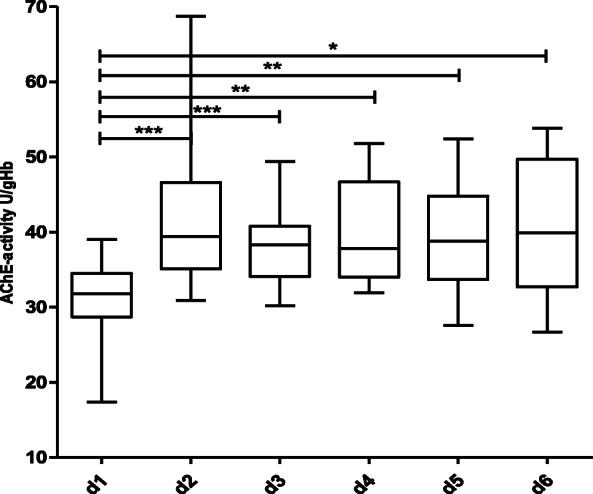
Course of AChE-activity over a period of 6 days in 15 septic patients with an increase of AChE-activity (CAM-ICU positive, differential diagnosis septic associated encephalopathy). Statistical significance was calculated using Wilcoxon matched-pairs test. *** *p* < 0.001, ** *p* < 0.01, * *p* < 0.05. Number of patients per day: d 1: *n* = 15, d 2: *n* = 15, d 3: *n* = 14, d 4: *n* = 12, d 5: *n* = 11, d 6: *n* = 10

**Fig. 5:**
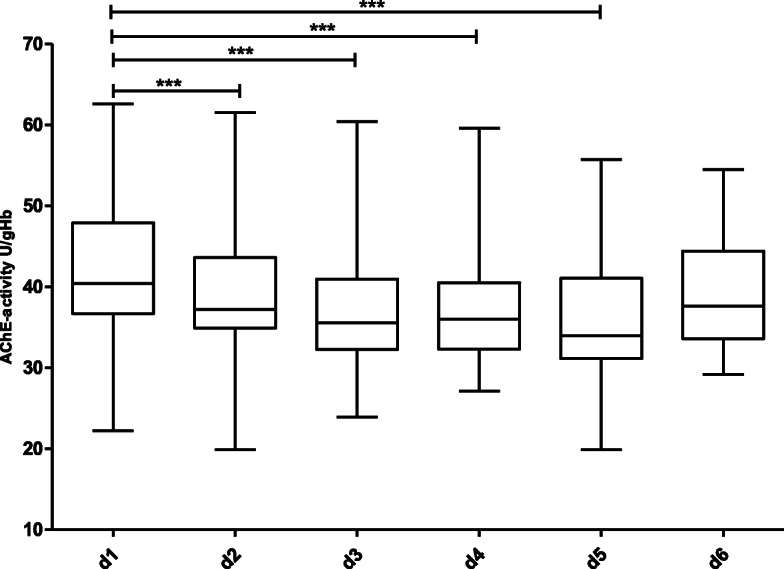
Course of AChE-activity in 30 septic patients with a decrease of AChE-activity (CAM-ICU positive, differential diagnosis septic associated encephalopathy (*n* = 27)), *n* = 3 permanently sedated until decease, n = 1 CAM-ICU negative. Statistical significance was calculated using Wilcoxon matched-pairs test. *** *p* < 0.001. Number of patients per day: d 1: *n* = 30, d 2: *n* = 30, d 3: *n* = 28, d 4: *n* = 27, d 5: *n* = 26, d 6: *n* = 22

In 15 of the 45 septic patients with suspected SAE (positive CAM-ICU) a statistically significant increase in AChE-activity from day 1 to day 6 could be demonstrated (Fig. 4). In 30 of the 45 septic patients with suspected SAE (positive CAM-ICU) a statistically significant decrease in AChE-activity from day 1 to day 5 could be demonstrated (Fig. 5).

#### Univariate analysis of septic patients

The univariate analysis showed a statistically significant increase or decrease in AChE-activity as a function of time in septic patients (period of at least 5 days after admission to the intensive care unit - AChE-activity decrease: *p* = 0.023, AChE-activity increase: *p* = 0.002). In contrast, no correlation of AChE-activity with age, gender, SAPS II, SOFA score, increase or decrease in AChE-activity, the occurrence of delirium/SAE or cognitive dysfunction could be demonstrated in septic patients.

In the non-septic patients neither a dependence of AChE-activity over time nor a correlation of AChE-activity with the parameters mentioned above could be demonstrated. Therefore, we refrained from demonstrating these results.

One statistically significant p-score between TISS-28 and septic patients with a decrease in AChE-activity (*p* = 0.041) was of no clinical significance, as the SAPS score showed no statistically significant difference (Table 2).

**Table 2:** Univariate analysis of septic patient subgroups

AChE-activity - independent variable	AChE-activity decrease			AChE-activity increase		
	Estimate	SE	*p*-value	Estimate	SE	*p*-value
**Time**	−0.57	0.25	**0.023**	1.44	0.45	**0.002**
**Age**	−0.04	0.09	0.674	−0.07	0.13	0.574
**Sex (male vs female)**	−0.37	2.82	0.896	1.31	4.71	0.782
**SAPS II**	0.14	0.09	0.144	0.08	0.17	0.608
**TISS-28**	0.42	0.20	**0.041**	0.14	0.22	0.522
**SOFA**	−0.20	0.52	0.703	−0.66	0.57	0.251
**Delir – DD SAE (yes vs no)**	−6.52	3.67	0.078	−1.40	4.65	0.764
**cognitive dysfunction (yes vs no)**	5.72	7.26	0.433	n.e.	n.e.	n.e.

Since the results of the univariate models exhibited no significance (type 1 error level was set to α = 5%), we did not run a more complex multivariable regression model afterwards.

## Discussion

The present study is the first to investigate the time-dependent increase or decrease in AChE-activity in septic patients with suspected SAE. Over a period of 5 consecutive days after admission to intensive care unit statistically significant changes occurred compared to baseline. In contrast, no change in AChE-activity was observed in non-septic patients even with delirium or cognitive impairment.

AChE-activity is downregulated due to a decrease of acetylcholine which is the substrate for acetylcholinesterase. However, despite a substrate deficiency AChE- activity can also be upregulated (see discussion below).

### Decrease of AChE-activity

In the present study, the time-dependent decrease in AChE-activity in the majority of septic patients with suspected SAE is consistent with the results of Bitzinger et al. They reported a significant and time-dependent decrease in AChE-activity in a rat cecal ligation puncture (CLP) - model [20]. In patients with sepsis and suspected SAE, inflammation appears to be a major cause for the alteration of cholinergic metabolism and thus AChE-activity. Sepsis leads to increased formation of oxygen radicals, which in turn can cause neuronal damage [33]. If neuronal damage affects cholinergic neurons, the transmitter acetylcholine is subsequently reduced and the activity of the surrogate parameter AChE-activity is altered. A deficiency of acetylcholine can manifest itself as delirium, i.e. attention and memory deficits [34]. Reduced concentrations of acetylcholine are also involved in the symptoms of Alzheimer’s disease. Thus, similarities with the pathophysiological changes in patients with SAE can be assumed. Méndez-Garrido et al. were able to show that in patients with Alzheimer’s disease, higher concentration of reactive oxygen species, e.g. H2O2, in central nervous system were detected. Oxidative stress decreases the AChE-activity and simultaneously increases the acetylcholine hydrolysis, which ultimately contributes to a central cholinergic deficiency [35].

### Increase of AChE-activity

In the present study, a time-dependent increase in AChE-activity was demonstrated in about one third of the septic patients with suspected SAE. In a CLP-induced sepsis model, the surviving mice showed a decrease in cholinergic neurons in the basal forebrain, a significant increase in AChE-activity and an increase in expression of their coding gene in the hippocampus and cortex, probably caused by microglial activation [22]. Enhanced AChE-activity leads to an increased breakdown of acetylcholine and ultimately to a cholinergic deficit, which is associated with characteristic symptoms such as memory disorders, disorientation, hypo- or hyperactivity [7, 8]. In this context of interest is the hippocampus as interface between short-term and long-term memory: changes in cholinergic transmission in the hippocampus of septic patients appear to play a central role in the pathogenesis of septic-associated encephalopathy. Many of the symptoms associated with SAE, such as memory disorders, attention deficits and consciousness disorders, can be attributed to changes in this particular area of the brain. Zivkovic et al. “identified the hippocampus as the site of dysfunction and pathology in sepsis induced delirium” by Magnetic Resonance Imaging (MRI) [19]. Given the possible pathophysiological changes postulated in SAE, both an increase and a decrease in AChE-activity seem plausible.

Considering the current evidence on the importance of esterase activities in patients with sepsis, most studies refer to changes in butyrylcholinesterase activity (BChE-activity), also called non-specific plasma esterase [32, 36–38]. Together with AChE-activity, BChE-activity is responsible for the maintenance and regulation of central cholinergic transmitter homeostasis. BChE-activity is subject to many different impacts and has therefore proven to be more of an outcome parameter [38, 39]. As a surrogate for the central transmitter status it is too inaccurate [40].

### Clinical-therapeutic relevance of AChE-activity in patients with suspected SAE

Until now, proof of efficacy of cholinesterase inhibitors for the treatment of delirium in critically ill patients could not be successfully demonstrated. However, in various experimental sepsis models an improved anti-inflammatory immune response was demonstrated by the administration of indirect parasympathopmimetic drugs crossing the BBB [39, 41–43]. Assuming that sepsis leads to increased permeability of the BBB, activation of microglia and damage to cholinergic neurons, the administration of indirect centrally acting parasympathomimetic drugs could have positive effects in patients with SAE. The ex-juvantibus administration of indirect parasympathomimetics which has been propagated up to now only leads to an improvement of cognitive symptoms in a few cases. Therefore, it seems to be crucial to identify those patients in whom a change in central cholinergic transmitter homeostasis is actually present as the cause of delirious symptomatology/SAE.

In summary, in septic patients with positive CAM-ICU and suspected SAE, there was a statistically significant increase or decrease in AChE-activity for at least 5 consecutive days compared to baseline. In contrast, in non-septic patients with delirium or cognitive dysfunction no statistically significant change in AChE-activity could be detected during the observation period. Therefore, in contrast to single measurements longitudinal measurement of AChE-activity in septic patients with delirium is able to diagnose SAE.

### Limitations

The present study has some limitations that need to be discussed. The study was conducted in an interdisciplinary surgical intensive care unit and planned as a prospective observational study. Two patient groups were distinguished (septic and non-septic patients) which differ considerably from each other in terms of group size and need for admission. Further subgroup analysis was not possible with the present case load.

A major limitation is the different sample size between the septic group and the non-septic group. However, in a prospective observational study, a different group size is not uncommon and the study plan ruled out an observation period longer than 12 months. Therefore, it was not possible to boost the case number. Due to this the calculated statistical power is 60% which may lead to underpowered results. To increase the statistical power up to 80% the sample size should have been 100 patients in each group. From previous studies it was known that a statistically significant change in AChE-activity if present could be postulated in septic patients also in small patient numbers [27]. If the statistically significant results of the present study are taken into account it can be assumed that the calculated power is not only 60% but underpowered results cannot be excluded with absolute certainty. Another point to discuss is the impossibility to define precisely the onset of sepsis in non-experimental studies. However, since sepsis can be classified into different phases it is conceivable that the increase or decrease in AChE-activity depends on the course of sepsis. The change in AChE-activity over the observation period may have been influenced also by other factors such as the application of anticholinergic drugs. Anticholinergic drugs (such as furosemide and opioids) have to be administered regularly and usually without alternative in ICU patients. In addition, SAE is a diagnosis of exclusion. Reliable diagnostic tools are missing. The CAM-ICU has been validated for the diagnosis of delirium but it is not known whether the high sensitivity and specificity also applies to patients with suspected SAE.

## Conclusion

So far, septic-associated encephalopathy is a diagnosis of exclusion. The pathophysiological changes underlying the SAE have not yet been adequately investigated. However, there is increasing evidence that changes in central cholinergic metabolism are at least partially responsible for the development and expression of SAE. Considering the present study results, repetitive measurement of AChE-activity is useful to detect changes in cholinergic transmitter homeostasis in patients with suspected SAE. AChE-activity is therefore suitable for differentiating SAE from other causes of delirium. In the future, changes in AChE-activity if detected over a period of several days may serve as rational basis for a targeted therapy using indirect parasympathomimetic drugs crossing the BBB, e.g. physostigmine, to improve delirious symptoms in SAE.

## Data Availability

The datasets used and/or analyzed during the current study are available from the corresponding author on reasonable request.

## Abbreviations

Ach: Acetylcholine
AChE-activity: Acetylcholinesterase-activity
BBB: Blood-brain barrier
BChE-activity: Butyrylcholinesterase-activity
CAM-ICU: Confusion assessment method for the intensive cate unit
ChE: Cholinesterase
CLP: Cecal ligation puncture
CNS: Central nervous system
CRP: C-reactive protein
EDTA: Ethylenediamine tetraacetic acid
ICU: Intensive Care Unit
MRI: Magnetic Resonance Imaging
PCT: Procalcitonin
RASS: Richmond agitation sedation scale
SAE: Septic associated encephalopathy
SAPS II: Simplified Acute Physiology Score
SOFA: Sequential organ failure assessment
TISS-28: Therapeutic Intervention Scoring System
